# Discordance between non-zero physician's global scores and absence of active joints in juvenile idiopathic arthritis: multicenter vs. single-center cohorts

**DOI:** 10.3389/fped.2026.1702667

**Published:** 2026-02-03

**Authors:** Ana Isabel Rebollo-Giménez, Silvia Rosina, Francesca Ridella, Silvia Maria Orsi, Elena Aldera, Marco Burrone, Valentina Natoli, Alessandro Consolaro, Francesca Bovis, Esperanza Naredo, Angelo Ravelli

**Affiliations:** 1 UOC Reumatologia e Malattie Autoinfiammatorie, IRCCS Istituto Giannina Gaslini, Genoa, Italy; 2 Department of Rheumatology, Gregorio Marañón University Hospital, Gregorio Marañón Health Research Institute (IiSGM), Madrid, Spain; 3 PhD Program in Medicine and Surgery, Autonomous University of Madrid (UAM), Madrid, Spain; 4 Dipartimento di Neuroscienze, Riabilitazione, Oftalmologia, Genetica e Scienze Materno-Infantili (DINOGMI), Università Degli Studi di Genova, Genoa, Italy; 5 Dipartimento di Scienze Della Salute (DISSAL), Università Degli Studi di Genova, Genoa, Italy; 6 Department of Rheumatology and Joint and Bone Research Unit, Fundación Jiménez Díaz University Hospital, Health Research Institute Fundación Jiménez Díaz (IIS-FJT, UAM), Madrid, Spain; 7 Direzione Scientifica, IRCCS Istituto Giannina Gaslini, Genoa, Italy

**Keywords:** clinimetric, juvenile idiopathic arthritis, outcomes, pediatric rheumatic diseases, physician global assessment

## Abstract

**Objective:**

This study aims to compare the frequency of instances in which the physician's global assessment of disease activity (PhGA) was scored >0 despite the absence of active joints in children with juvenile idiopathic arthritis (JIA), using two multicenter patient datasets and one single-center dataset from a pediatric rheumatology center with expertise in clinimetric assessments.

**Methods:**

Data were extracted from two multicenter datasets and one single-center dataset, comprising 9,081, 563, and 394 patients, respectively. Patients with an active joint count (AJC) of 0 were included. The PhGA and fulfillment of other criteria from the 2004 or 2011 Wallace definition of clinically inactive disease (CID) were assessed. UpSet plots were used to analyze the frequency and overlap of PhGA and CID items across the datasets.

**Results:**

Among patients with an AJC of 0, the percentage for whom a PhGA score >0 was the sole unmet CID criterion was 14.8% and 13.7% in the two multicenter datasets and 5.1% in the single-center dataset. The CID criteria that were most frequently not met when the PhGA was scored >0 were elevated acute-phase reactants (APRs) and morning stiffness lasting ≥15 min.

**Conclusion:**

The discordance between the absence of active joints and a PhGA score >0 was less common in the single-center sample, suggesting that regular use and training may increase concordance between PhGA and AJC in patients without clinical signs of joint disease. APR elevation and parent-/patient-reported morning stiffness seemed to play a major role in prompting physicians to assign a non-zero global score.

## 1 Introduction

The physician's global assessment of disease activity (PhGA) is a cardinal outcome measure of juvenile idiopathic arthritis (JIA). It captures the evaluator's subjective estimation of the patient's disease activity at the time of the visit and integrates information from the clinical history with the physical examination findings, laboratory test results, and other available investigations. The PhGA is rated on a 10-cm linear visual analog scale (VAS) or a 21-circle numeric rating scale (NRS) (1).

The PhGA has been found to possess strong responsiveness to clinical important change (2), to be a reliable indicator of overall disease activity in all stages of the illness (3), to represent a suitable gold standard in validation analysis of newly developed outcome measures (4), and to predict disease outcomes in JIA (5). Based on its good measurement properties, this tool has been incorporated into endpoints used to assess therapeutic response (6) and disease activity states (7–11) and is widely used to quantify the level of disease activity in both clinical practice and observational studies.

Owing to recent therapeutic advances, current clinical practice requires achieving disease remission in all patients with JIA. The recommendations for the treat-to-target (T2T) strategy in JIA identify clinically inactive disease (CID) as the primary therapeutic goal (12). Because the PhGA is included in all measurements used to assess CID, it must be rated accurately. However, concerns have been raised by recent observations of wide variability in PhGA scores among pediatric rheumatologists (13). Furthermore, it has been reported that clinicians sometimes record a non-zero PhGA despite the absence of active joints and fulfillment of other CID criteria, which reflects the intended use of the PhGA as a global judgment that may capture disease aspects beyond joint counts (14, 15). This observation has led to modifying the CID criteria in some therapeutic studies by setting a minimum PhGA score of 1 (16) or even 2 instead of zero (17). This issue is compounded by the observation that the PhGA score may be influenced by patient symptoms unrelated to inflammation (18).

Until recently, there were no guidelines aimed at standardizing PhGA scoring by designating the elements that should be valued and integrated in its assessment. In 2024, consensus-based recommendations for scoring the PhGA in non-systemic and systemic JIA were developed through a multinational collaborative effort (19).

Because it remains unclear whether training improves concordance among clinicians in rating the PhGA in patients with CID, we compared, by means of a descriptive and exploratory analysis, the frequency of visits in which the assessor assigned a PhGA >0 despite the absence of joints with active arthritis across two multicenter patient datasets and a cohort from a single pediatric rheumatology center with established tradition and expertise in clinimetric assessment.

## 2 Methods

### 2.1 Study design and patient selection

Data were extracted from three datasets (two multicenter and one single-center) of patients with JIA (20). The first multicenter dataset included 9,081 patients recruited from 130 centers in 49 countries and assessed cross-sectionally as part of a survey of the epidemiology, treatment, and outcomes of JIA (the EPOCA study) (21). Patients were enrolled between 2011 and 2016. This study data were utilized in a previously published analysis investigating the drivers of non-zero PhGA scores in patients with no active joints (15).

The second multicenter dataset comprised 563 patients with systemic JIA (sJIA) drawn from two studies. The first study, conducted between February 2017 and December 2018, enrolled patients from 27 centers of the Italian Pediatric Rheumatology Study Group and 16 international pediatric rheumatology centers located in regions with a high prevalence of sJIA, with the aim of developing and validating the systemic Disease Activity Score (sJADAS) (22). The second study, carried out from February to November 2022, recruited patients across 30 pediatric rheumatology centers in 11 countries and focused on defining criteria for disease activity states based on the sJADAS (11). For the present analysis, patient cohorts from both studies were combined.

The single-center dataset comprised 394 patients followed at the Gaslini Institute of Genoa, Italy, who were part of a study investigating the percentage of patients who achieved CID in the decade preceding the publication of the T2T recommendations for JIA (23). All patients were evaluated within the first 6 months after disease onset between 2007 and 2017.The Gaslini Institute is a large tertiary care pediatric hospital that hosts a pediatric rheumatology group, which has historically been engaged in the development and regular application of clinical outcome measures and in training in clinimetric assessments.

Because the second and third datasets included one or more follow-up visits, we included only one visit per patient in the analysis. Specifically, among visits with an active joint count (AJC) of 0, we selected either the last visit or the visit with the most complete set of CID criteria available. Both the PhGA and AJC were assessed by treating clinicians as part of routine clinical care. No blinding was introduced, and differences in training or standardization across centers were not formally assessed. Any differences between datasets are, therefore, described without causal interpretation.

### 2.2 Outcome measures

The PhGA was rated for all patients using a 21-circle NRS, ranging from 0 (no activity) to 10 (maximum activity) (1). Joint assessment was made by the treating physician, who recorded—across the 73 joints included in the standard articular examination—the presence of swelling, tenderness/pain on motion, and limitation of range of motion, as previously reported (24). A joint was defined as active if it displayed swelling or, when swelling was absent or not detectable, if pain/tenderness was accompanied by restricted motion (25).

In addition to the PhGA and the count of active joints, other components of the 2004 (7) or 2011 (8) definition of CID, depending on its applicability in the study datasets, were assessed. According to the 2011 definition, a patient is classified as having CID when all the following conditions are met: (1) no active joints, (2) no systemic manifestations attributable to JIA, (3) no active uveitis; (4) normal acute-phase reactants (APRs), (5) a PhGA indicating no disease activity, and (6) morning stiffness lasting ≤15 min. The 2004 definition includes the same criteria but lacks the sixth item.

### 2.3 Statistical analysis

For each dataset (EPOCA, sJADAS, and Gaslini), results were summarized as absolute frequencies and percentages. Differences in these frequencies among the three datasets were assessed using chi-square tests or Fisher's exact tests when expected cell counts were <5. When the overall test indicated a significant difference, pairwise *post-hoc* comparisons between datasets were conducted using chi-square or Fisher's exact tests, with Bonferroni correction for multiple testing.

To assess fulfillment and overlap of CID criteria within each patient cohort, we generated UpSet plots (26), a visualization technique that captures intersections among multiple categorical variables. To generate UpSet plots of the distribution of patients who meet or do not meet CID criteria, a structured, data-driven approach was applied. First, a binary dataset was constructed in which each row represented an individual patient and each column corresponded to one of the criteria that define CID. Criteria were encoded as 1 if met and 0 if not met, which facilitated the identification of unique patient subsets defined by the specific criteria that were not met. The dataset was then transformed into a format suitable for UpSet visualization, with each unique combination of unmet criteria mapped to a corresponding patient count.

## 3 Results

Among patients/visits with complete CID data available, the proportion of those with an AJC of 0 was 50% (3,630/7,265) in the EPOCA dataset, 60.7% (327/539) in the sJADAS dataset, and 70.5% (292/414) in the Gaslini dataset. The frequency of CID was assessed by the 2011 criteria (8) in the first dataset and by the 2004 criteria (7) in the other two datasets.

The frequency of fulfillment or non-fulfillment of individual or combined CID criteria across the three patient cohorts is shown in Table 1. The percentage of patients who met all CID criteria was higher in the Gaslini dataset (67.9%) than in the EPOCA and sJADAS datasets (46.3% and 37.3%, respectively; *p* < 0.0001). The proportion of patients with PhGA >0 (with all other CID criteria met) as the sole unmet CID criteria was lower in the Gaslini dataset (5.1%) than in the EPOCA and sJADAS datasets (14.8% and 13.7%, respectively; *p* < 0.0001). Compared with the two multicenter cohorts, the Gaslini cohort also showed a lower frequency of patients with PhGA >0 plus one or more other CID criteria not met (4.8% vs. 18.2% and 29.7%, respectively; *p* < 0.0001). In contrast, the proportion of patients with PhGA = 0 but one or more other CID criteria not met was comparable across the three datasets (20.7%, 19.3%, and 22.6%, respectively; *p* = 0.656).

**Table 1:** Frequency of individual and combined CID items in patients judged by the treating physician as having no active joints.^a^

Items	EPOCA study^b^ (*n* = 3,630)	sJADAS study^c^ (*n* = 327)	Gaslini cohort^c^ (*n* = 292)	*p*-Value
Patients meeting all CID criteria	1,680 (46.3)	122 (37.3)	198 (67.9)	<0.0001
Patients with only PhGA >0	536 (14.8)	45 (13.7)	15 (5.1)	<0.0001
Patients with PhGA >0 and ≥1 other non-met ID criteria	662 (18.2)	97 (29.7)	14 (4.8)	<0.0001
Patients with PhGA = 0 and ≥1 other non-met ID criteria	752 (20.7)	63 (19.3)	65 (22.6)	0.656
Patients with PhGA >0 and only elevated APR	199 (5.5)	64 (19.6)	8 (2.7)	<0.0001
Patients with PhGA >0 and only active uveitis	35 (1.0)	−	4 (1.4)	0.531
Patients with PhGA >0 and only active systemic features	10 (0.3)	4 (1.2)	0 (0)	0.001
Patients with PhGA >0 and only morning stiffness ≥ 15 min	277 (7.6)	−	−	−

CID, clinically inactive disease; PhGA, physician's global assessment of overall disease activity; APR, acute phase reactants.

a Data are the numbers (percentages).

b CID was assessed by the 2011 Wallace definition.

c CID was assessed by the 2004 Wallace definition.

The percentage of patients with PhGA >0 and elevated APR, with all other CID criteria met, was higher in the sJADAS dataset (19.6%) than in the EPOCA and Gaslini datasets (5.5% and 2.7%, respectively; *p* < 0.0001). The percentage of patients with PhGA >0 and active uveitis, with all other CID criteria met, was low and comparable in the EPOCA and Gaslini datasets (1% and 1.4%, respectively; *p* = 0.531). Likewise, the proportion of patients with PhGA >0 and active systemic features, with all other CID criteria met, was low and similar across the three datasets (0.3%, 1.2%, and 0%, respectively), although the overall difference was statistically significant (*p* = 0.001). In the EPOCA dataset, 7.6% of the patients had PhGA >0 and morning stiffness ≥15 min as the sole other unmet CID criterion.

The frequency of individual and combined CID items among patients not meeting CID criteria but judged by treating physicians as having no active joints is given in Supplementary Table S1. This analysis shows that physicians of the Gaslini team assigned a PhGA >0 less frequently when they did not detect any active joint, irrespective of the presence of other non-met CID criteria, and assigned a PhGA = 0 more frequently under the same circumstances.

The comparison of the frequency of individual and combined CID components among patients judged by the treating physician as having no active joints across the study datasets is presented in Supplementary Table S2. All differences were statistically significant, except for the comparison of patients with only PhGA >0 between the EPOCA and sJADAS datasets, the comparison of patients with only elevated APR between the EPOCA and Gaslini datasets, and the comparison of active systemic features across all three datasets. These findings underscore the widespread diversity in assessing CID criteria across physicians and clinical settings.

**Figure 1:**
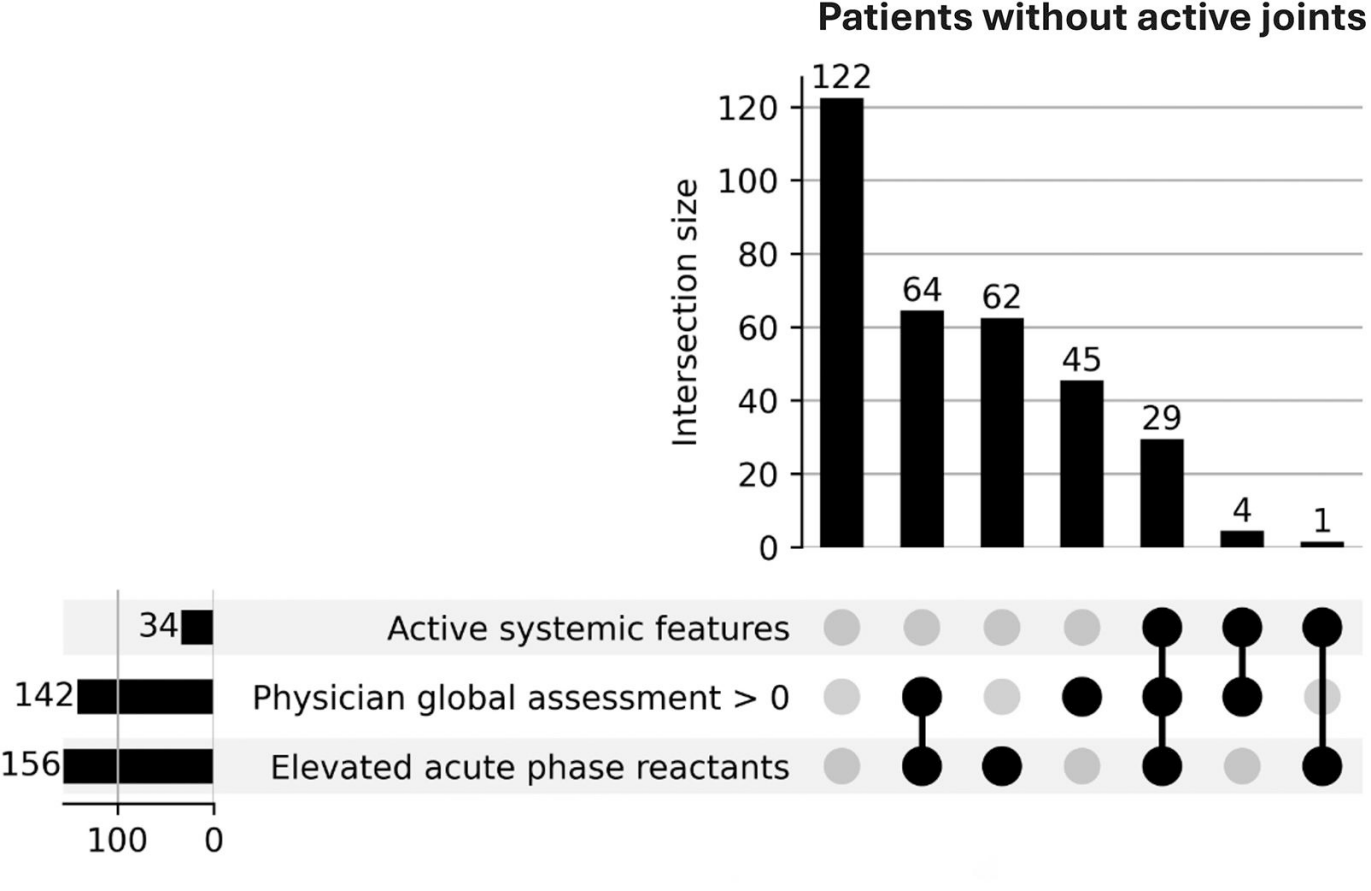
sJADAS dataset—UpSet plot showing distinct combinations of items of the 2004 clinically inactive disease definition ranked by frequency in patients with no active joints.

**Figure 2:**
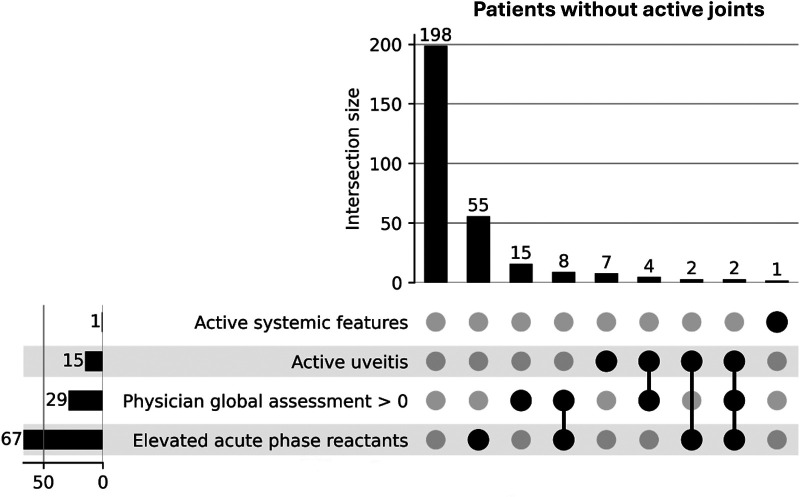
Gaslini dataset—UpSet plot showing distinct combinations of items of the 2004 clinically inactive disease definition ranked by frequency in patients with no active joints.

UpSet plots depicting the distinct combinations of items of CID criteria in the sJADAS and Gaslini datasets are shown in Figures 1, 2, respectively. The UpSet plot for the EPOCA dataset has been included in a previous publication (15).

In the UpSet plot for the EPOCA dataset reported by Alongi et al. (15), the most frequent single reason for not meeting the CID definition was a PhGA score >0 (14.8%), followed by the elevation of acute-phase reactants (10.8%), morning stiffness lasting ≥15 min (7.1%), and the presence of uveitis (1%). The most common combination of criteria that led to not fulfilling the CID definition was PhGA >0 plus morning stiffness lasting ≥15 min (7.6%), followed by PhGA >0 plus elevated APR (5.5%) and PhGA >0 plus morning stiffness lasting ≥15 min and elevated APR (2.9%). Similar findings were obtained when we reanalyzed these data using the 2004 CID criteria instead of the 2011 CID criteria, i.e., after excluding the morning stiffness ≥15 min criterion (Supplementary Figure S1).

In the UpSet plot for the sJADAS dataset (Figure 1), which included only patients with systemic JIA, the most frequent single reason for not fulfilling the CID definition was elevated acute-phase reactants (19%), followed by PhGA >0 (13.8%). The most common combination of criteria that led to not meeting the CID definition was PhGA >0 plus elevated APR (19.6%), followed by PhGA >0 plus elevated APR and active systemic features (8.9%) and PhGA >0 plus active systemic features (1.2%).

To enable comparison with patients in the sJADAS dataset, UpSet plots were generated for the subset of sJIA patients included in the EPOCA study, assessed using either the 2004 and 2011 CID criteria. The results obtained in the two samples were overall similar; however, in the EPOCA cohort, the most frequent single reason for not fulfilling both 2004 and 2011 CID definitions was PhGA >0, followed by elevated APRs. As for the sJADAS dataset, the most common combination of criteria that led to not meeting both CID definitions was PhGA >0 plus elevated APRs (Supplementary Figures S2, S3).

In the UpSet plot for the Gaslini dataset (Figure 2), the most frequent single reason for not fulfilling the CID definition was elevated acute-phase reactants (18.8%), followed by PhGA >0 (5.1%) and active uveitis (2.4%). The most common combination of criteria that led to not meeting the CID definition was PhGA >0 plus elevated APRs (2.7%), followed by PhGA >0 plus active uveitis (1.4%), active uveitis plus elevated acute-phase reactants (0.7%), and PhGA >0 plus elevated APRs and active uveitis (0.7%).

## 4 Discussion

In this descriptive and exploratory analysis, we compared the percentage of instances in which physicians assigned a PhGA score >0 despite detecting no active joints across two multicenter patient cohorts and one single-center sample. The single-center patients were evaluated at a tertiary care facility (the Gaslini Institute), which is traditionally engaged in developing outcome measures and providing training in clinimetric assessments. Altogether, the enrolled patients are likely representative of all phenotypes of JIA encountered in pediatric rheumatology clinics worldwide. Analysis of CID components other than the PhGA and AJC was undertaken to obtain insights into factors that may contribute to discordance between these two measures.

Our analysis confirms previous observations that many clinicians do not score their PhGA as zero despite detecting no joints with active disease (15). However, we found that this phenomenon occurred less frequently in the single-center sample than in the two multicenter datasets. This disparity was observed both when the PhGA >0 was the sole unmet CID criterion and when PhGA >0 was accompanied by the lack of fulfillment of one or more additional CID criteria. These findings indicate that physicians of the Gaslini team placed greater value on the state of joint disease than on the presence of the other CID features when scoring their PhGA. Importantly, the higher rate of CID in the Gaslini cohort compared to the other samples reflects good performance of these two measures in patients with a favorable disease outcome.

Among CID criteria other than the AJC and PhGA, the item that most frequently co-occurred with PhGA >0 was morning stiffness lasting ≥ 15 min. Although this parameter was available only in the EPOCA dataset, this observation suggests that the parent-/patient-reported persistent disease activity prompts many physicians not to score their PhGA as zero. In a recent multinational survey, duration of morning stiffness was selected as one of the factors influencing the PhGA in non-systemic JIA by more than half of respondents (27).

Elevation of APRs ranked second after morning stiffness in affecting physicians’ judgments in the EPOCA dataset, and it had a major impact in the sJADAS dataset, which comprised only patients with sJIA, as well as in the subset of sJIA patients within the EPOCA dataset. It seems, therefore, that many physicians value substantial weight on elevated APR when assigning a non-zero PhGA score despite the absence of active joints in patients with sJIA. Inflammation biomarkers have been found to play a leading role in affecting PhGA scoring in patients with sJIA (27).

The isolated presence of active uveitis accounted for discordance between the PhGA and AJC in approximately 1% of instances in the EPOCA and Gaslini datasets. In contrast, PhGA >0 in association with only active systemic manifestations was observed in a negligible number of cases across all three datasets. The latter observation is likely explained by the rare occurrence of active systemic manifestations in the absence of concomitant elevation of APRs.

Analysis of the UpSet plots confirmed that the most common reasons physicians did not assign a PhGA score of 0 despite the absence of active joints were morning stiffness lasting ≥15 min (in the EPOCA dataset) and elevated APRs, especially in the sJADAS dataset and in the subset of sJIA patients within the EPOCA dataset. An isolated increase in APR accounted for failure to meet CID criteria in a sizeable proportion of patients in the sJADAS, sJIA EPOCA, and Gaslini datasets. Because APRs may be influenced by factors external to the disease, the impact of this phenomenon on CID estimation deserves further investigation.

Recent studies suggest that implementing structured training programs and collaborative initiatives can enhance reliability, improve disease assessments, and optimize treatment decisions, paving the way for more consistent and effective disease management practices. Buckley et al. (28) reported significant reductions in clinical JADAS10 scores among patients with polyarticular JIA after implementing a program that trained physicians in standardized disease activity measurement. Harris et al. (29) described a collaborative learning health system approach that resulted in significant improvements in disease activity metrics.

Our results should be interpreted in light of some potential limitations. We recognize that the study relied on pre-existing datasets that were not originally designed to address this specific question. The number of evaluators in the EPOCA and sJADAS datasets was greater than in the Gaslini dataset, which involved only 8–10 clinicians. Furthermore, the size of the patient population was much larger in the EPOCA dataset than in the two other datasets. These numerical disparities might be responsible for the higher frequency of divergent assessments observed in the multicenter samples. We must also acknowledge that by highlighting the different degrees of discordance between evaluators across the three patient samples, we cannot imply that the assessments made at the Gaslini Institute are correct or that a PhGA score of 0 is equivalent to an AJC of 0. Due to the inherent heterogeneity of multicenter datasets, we deliberately restricted our analysis to a descriptive characterization of observed patterns and avoided causal inferences. We did not perform multivariable modeling to adjust for potential confounders, such as disease duration, treatment exposure, joint damage, or center-level practice patterns. The omission of factors like non-inflammatory pain, physical function, and quality of life limits our ability to fully understand why physicians score PhGA >0 when AJC = 0 and may confound the apparent impact of clinimetric training. Morning stiffness lasting ≥15 min, a key driver of discordance in EPOCA, was not available or applicable in the other datasets, which prevents a cross-cohort comparison of this important patient-reported symptom. The multicenter datasets likely include substantial between-center variability in practice, training, and patient characteristics. Because we lack measures of center-specific clinimetric training, we acknowledge this gap as a limitation to prevent overinterpretation of our findings.

In conclusion, this study confirms previous observations that a sizeable proportion of physicians assign a PhGA score >0 in patients without clinical evidence of inflammation in any joint. This disparity was less pronounced in patients evaluated in a single center with a well-established tradition and expertise in clinimetric assessment. However, our findings do not imply that the PhGA should reflect only the AJC. Rather, the observed disparity between PhGA >0 and AJC = 0 means that many physicians not only value the absence of active joints but also consider other disease-related factors in judging whether the patient is in the state of CID. Our results should be compared with those of other studies conducted in different patient populations, diverse contexts, and conducted prospectively with a predefined methodology.

## Data Availability

The raw data supporting the conclusions of this article will be made available by the authors without undue reservation.
