# Supplementary material for: Discordance between non-zero physician's global scores and absence of active joints in juvenile idiopathic arthritis: multicenter vs. single-center cohorts
Source: Front Pediatr. 2026 Feb 3;14:1702667. doi: 10.3389/fped.2026.1702667 (PMC12909535; doi:10.3389/fped.2026.1702667)

## Supplementary Table S1. Frequency of individual and combined CID items in patients not meeting all CID criteria, but judged by the treating physician as having no active joints*

|  | EPOCA study  (n = 1950) | sJADAS study  (n = 205) | Gaslini cohort  (n = 94) | p-value |
| --- | --- | --- | --- | --- |
| Patients with only PhGA > 0 | 536 (27.5%) | 45 (22.0%) | 15 (16.0%) | 0.014 |
| Patients with PhGA > 0 and ≥ 1 other non-met ID criteria | 662 (33.9%) | 97 (47.3%) | 14 (14.9%) | <.0001 |
| Patients with PhGA = 0 and ≥ 1 other non-met ID criteria | 752 (38.6%) | 63 (30.7%) | 65 (69.1%) | <.0001 |
| Patients with PhGA > 0 and only elevated APR | 199 (10.2%) | 64 (31.2%) | 8 (8.5%) | <.0001 |
| Patients with PhGA > 0 and only active uveitis | 35 (1.8%) | – | 4 (4.3%) | 0.101 |
| Patients with PhGA > 0 and only active systemic features | 10 (0.5%) | 4 (2.0%) | 0 (0.0%) | 0.058 |
| Patients with PhGA > 0 and only morning stiffness ≥ 15 min. | 277 (14.2%) | ~~–~~ | ~~–~~ | ~~–~~ |

*Data are the number (percentage). CID was assessed by 2004 Wallace definition.

CID = clinically inactive disease; PhGA = physician’s global assessment of overall disease activity; APR = acute phase reactant

**Supplementary Table S2.** Comparison of the frequency of individual and combined CID items in patients judged by the treating physician as having no active joints between EPOCA, sJADAS, and Gaslini datasets.

|  | **Comparison** | **P-value** | **P-adj_Bonf** |
| --- | --- | --- | --- |
| Patients who met all CID criteria | EPOCA vs sJADAS | 0.002 | 0.005 |
| Patients who met all CID criteria | EPOCA vs Gaslini | <.0001 | <.0001 |
| Patients who met all CID criteria | sJADAS vs Gaslini | <.0001 | <.0001 |
| Patients with only PhGA > 0 | EPOCA vs sJADAS | 0.623 | 1.000 |
| Patients with only PhGA > 0 | EPOCA vs Gaslini | <.0001 | <.0001 |
| Patients with only PhGA > 0 | sJADAS vs Gaslini | <.0001 | 0.001 |
| Patients with PhGA > 0 and ≥ 1 other non-met CID criteria | EPOCA vs sJADAS | <.0001 | <.0001 |
| Patients with PhGA > 0 and ≥ 1 other non-met CID criteria | EPOCA vs Gaslini | <.0001 | <.0001 |
| Patients with PhGA > 0 and ≥ 1 other non-met CID criteria | sJADAS vs Gaslini | <.0001 | <.0001 |
| Patients with PhGA > 0 with/without ≥ 1 other non-met CID criteria | EPOCA vs sJADAS | <.0001 | 0.000 |
| Patients with PhGA > 0 with/without ≥ 1 other non-met CID criteria | EPOCA vs Gaslini | <.0001 | <.0001 |
| Patients with PhGA > 0 with/without ≥ 1 other non-met CID criteria | sJADAS vs Gaslini | <.0001 | <.0001 |
| Patients with PhGA > 0 and only elevated APR | EPOCA vs sJADAS | <.0001 | <.0001 |
| Patients with PhGA > 0 and only elevated APR | EPOCA vs Gaslini | 0.044 | 0.131 |
| Patients with PhGA > 0 and only elevated APR | sJADAS vs Gaslini | <.0001 | <.0001 |
| Patients with PhGA > 0 and only active systemic features | EPOCA vs sJADAS | 0.024 | 0.071 |
| Patients with PhGA > 0 and only active systemic features | EPOCA vs Gaslini | 1.000 | 1.000 |
| Patients with PhGA > 0 and only active systemic features | sJADAS vs Gaslini | 0.126 | 0.379 |

CID = clinically inactive disease; PhGA = physician’s global assessment of overall disease activity; APR = acute phase reactants; P adj bonf= p-values adjusted according to the Bonferroni method.

**Supplementary Figure S1.** EPOCA dataset - UpSet plot showing distinct combinations of items of 2004 clinically inactive disease criteria ranked by frequency in patients with no active joints.


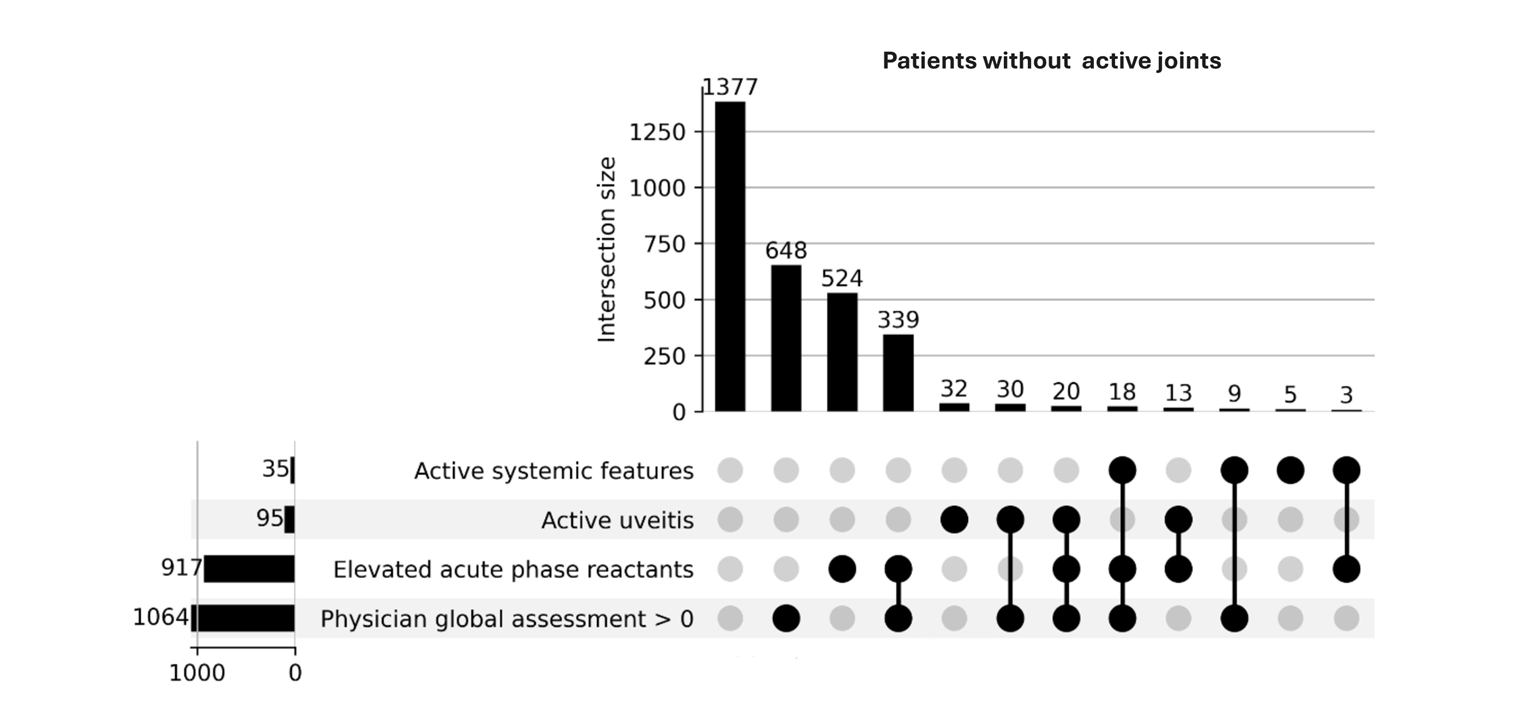


**Supplementary Figure S2.** EPOCA dataset - UpSet plot showing distinct combinations of items of 2011 clinically inactive disease criteria ranked by frequency in the subset of patients with systemic juvenile idiopathic arthritis and no active joints.


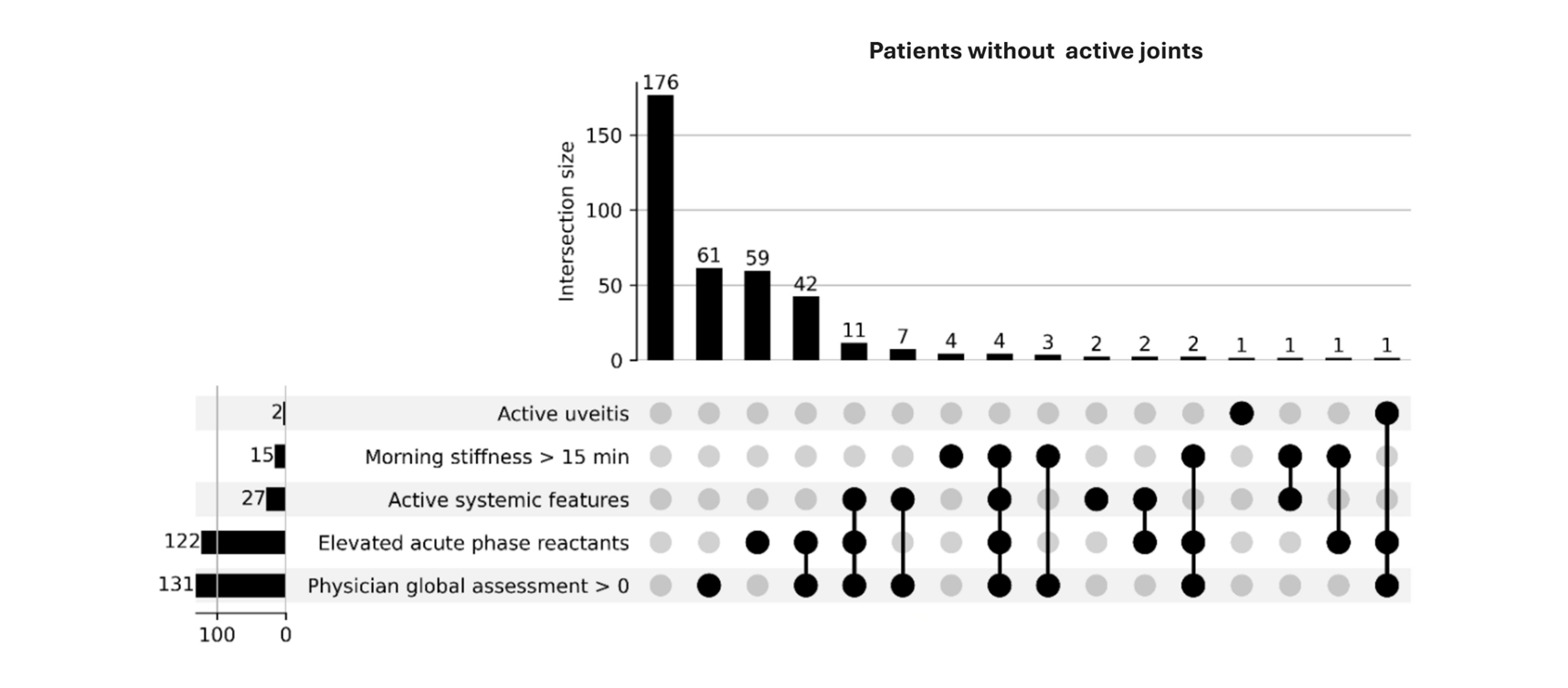


**Supplementary Figure S3.** EPOCA dataset - UpSet plot showing distinct combinations of items of 2004 clinically inactive disease criteria ranked by frequency in the subset of patients with systemic juvenile idiopathic arthritis and no active joints.


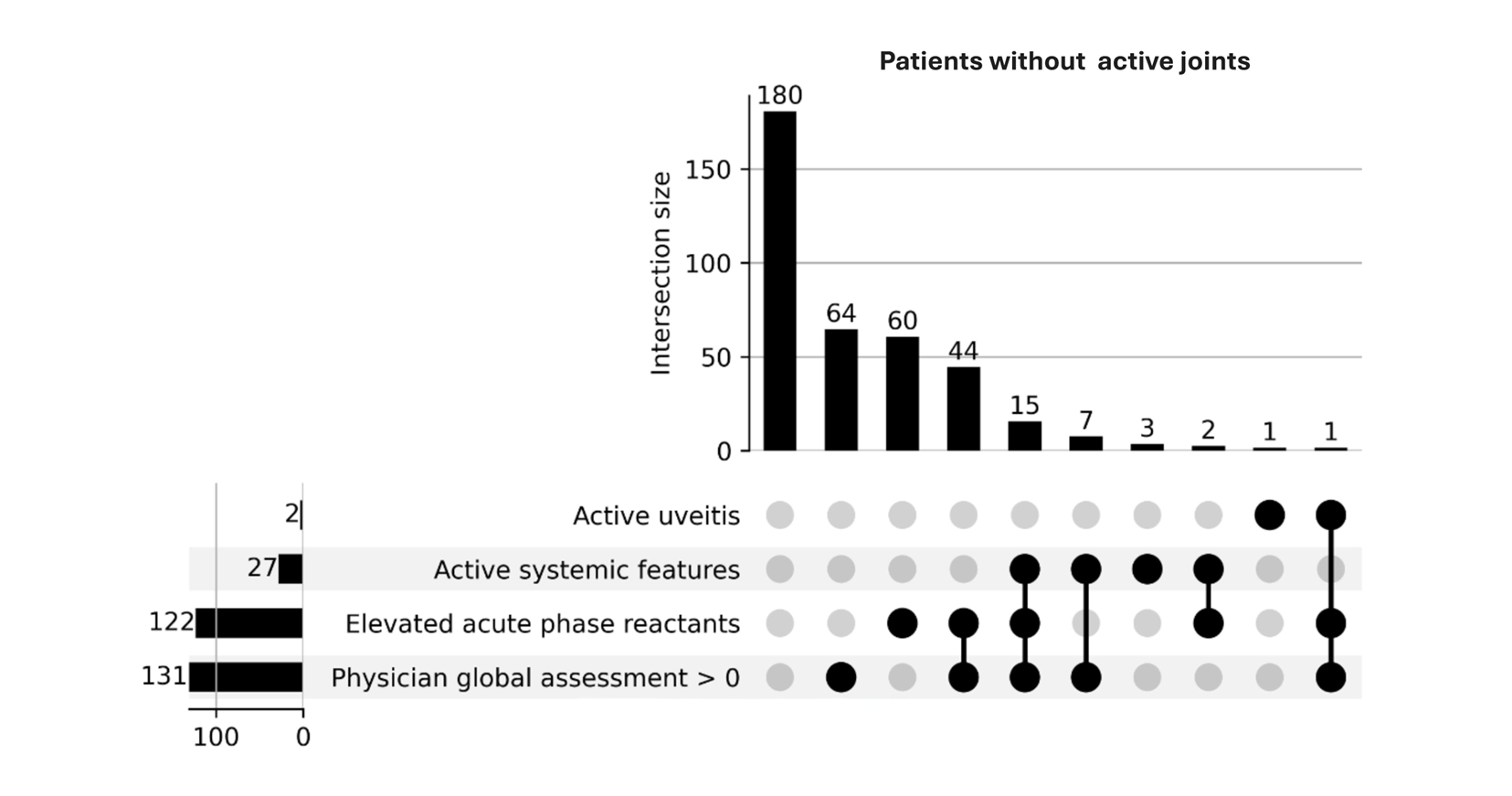

Supplement: Supplementary file 1 [file Supplementaryfile1.docx]
